# Effects of quercetin supplementation on the quality and metabolomic characteristics of sex-sorted frozen semen from Australian Wagyu bulls

**DOI:** 10.3389/fvets.2026.1951637

**Published:** 2026-09-28

**Authors:** Chao Yuan, Yuefu Yin, Xin Wang, Bo Yu, Junda Wu, Yuanfeng Zhao, Jing Liu, Kaikai Zhang, Wenzhang Zhou, Yongcai Zhu, Longxin Xu

**Affiliations:** 1 Institute of Animal Husbandry and Veterinary Science, Guizhou Academy of Agricultural Sciences, Guiyang, Guizhou, China; 2 Guizhou Provincial Key Laboratory of Livestock and Poultry Genetic Resources Innovation and Utilization, Guiyang, Guizhou, China

**Keywords:** Australian Wagyu bull, cryopreservation, lipid metabolism, oxidative stress, quercetin, sex-sorted semen, sperm quality, untargeted metabolomics

## Abstract

This study aimed to evaluate the effects of different concentrations of quercetin on the post-thaw sperm quality of sex-sorted frozen semen from Australian Wagyu bulls and to investigate the underlying metabolic regulatory mechanisms using untargeted metabolomics. Sex-sorted frozen semen from Australian Wagyu bulls was treated with 0, 10, 50, 100, 150, and 200 μM quercetin. Sperm motility parameters, plasma membrane integrity, acrosome integrity, mitochondrial membrane potential, DNA integrity, reactive oxygen species (ROS) levels, and antioxidant-related indices were assessed. Metabolomic profiling was further performed to compare the 50 μM quercetin-treated group with the control group. The results showed that the 50 μM quercetin group exhibited better sperm viability, motility, straight-line velocity (VSL), curvilinear velocity (VCL), and average path velocity (VAP), together with reduced ROS levels and increased activities of antioxidant enzymes, including SOD, CAT, and GSH-Px. High concentrations of quercetin (≥150 μM) adversely affected sperm motility and several functional parameters. Metabolomic analysis revealed clear metabolic differences between the 50 μM quercetin-treated group and the control group, with 131 upregulated and 395 downregulated differential metabolites identified. KEGG enrichment analysis indicated that these differential metabolites were mainly involved in lipid-related pathways, including glycerophospholipid metabolism and arachidonic acid metabolism. These results suggest that an appropriate concentration of quercetin may improve the post-thaw quality of sex-sorted frozen semen from Australian Wagyu bulls by alleviating oxidative stress and regulating lipid metabolism. This study provides experimental evidence and metabolomic insights for optimizing cryoprotectant formulations for bovine sex-sorted frozen semen.

## 1 Introduction

With the global beef cattle industry moving toward greater efficiency, precision, and intensive utilization of genetic resources, improving the use efficiency of the genetic potential of elite breeding bulls has become an important research focus in modern livestock breeding systems (1). Sex-sorted frozen semen technology enables directional control of offspring sex and has substantial advantages in improving the utilization efficiency of high-quality females, optimizing herd structure, and increasing economic returns; therefore, it has been widely applied in dairy cattle and high-value beef cattle breeding systems (2). However, compared with conventional frozen semen, sex-sorted frozen semen is more vulnerable to multiple stressors during flow cytometric sorting, cryopreservation, and thawing, including mechanical stress, osmotic changes, cold shock, and ice crystal formation. These stressors can impair sperm membrane structure, disrupt mitochondrial function, reduce DNA integrity, and decrease fertilizing capacity, thereby limiting the broader application of this technology (3, 4).

Oxidative stress is one of the key mechanisms underlying sperm cryoinjury. The freeze–thaw process can induce excessive accumulation of reactive oxygen species (ROS), which further causes lipid peroxidation, reduced membrane fluidity, and mitochondrial dysfunction (5). Because mammalian sperm membranes are rich in polyunsaturated fatty acids, they are particularly susceptible to oxidative damage. Excessive ROS not only damages the sperm plasma membrane and acrosomal structure but also causes DNA damage and impairs ATP production, ultimately weakening sperm motility and fertilizing potential (6). Therefore, supplementation of freezing extenders with appropriate antioxidants has become an important strategy for improving frozen semen preservation by alleviating oxidative stress and maintaining sperm functional stability.

Quercetin is a naturally occurring flavonoid widely present in plants and has strong free radical-scavenging activity, anti-inflammatory effects, and membrane-protective properties (7). In recent years, quercetin has attracted increasing attention in the field of cryoprotection of animal reproductive cells. Previous studies have shown that appropriate concentrations of quercetin can improve post-thaw sperm motility, membrane integrity, and antioxidant status in species such as pigs, horses, goats, and buffaloes, and can also reduce cryopreservation-induced DNA damage to some extent (8, 9). Mechanistically, quercetin may protect cryopreserved spermatozoa by scavenging excessive ROS, limiting lipid peroxidation of polyunsaturated-fatty-acid-rich membranes, and helping preserve mitochondrial, plasma-membrane, acrosomal, and DNA integrity during freeze–thaw stress.

In addition to conventional sperm quality indicators, metabolomics provides a new perspective for elucidating the mechanisms of sperm cryoinjury. Metabolomics can systematically reflect small-molecule metabolic changes in cells under specific physiological or stress conditions and helps reveal associations between metabolic pathways and sperm function (10). In recent years, several metabolites associated with fertility, freezability, and sperm motility have been identified in bovine seminal plasma and sperm samples, among which lipid metabolism, energy metabolism, and redox homeostasis are considered important metabolic foundations affecting frozen semen quality. In particular, pathways such as glycerophospholipid metabolism, unsaturated fatty acid metabolism, and arachidonic acid metabolism are closely related to sperm membrane stability, ROS generation, and membrane lipid peroxidation (11–13). However, few studies have investigated how natural antioxidants improve the quality of bovine sex-sorted frozen semen through metabolic remodeling, and the related metabolic regulatory mechanisms remain unclear.

Based on this background, the present study used sex-sorted frozen semen from Australian Wagyu bulls as the experimental material and supplemented freezing extenders with different concentrations of quercetin. The effects of quercetin on post-thaw sperm motility, membrane integrity, mitochondrial membrane potential, DNA integrity, and oxidative stress-related indices were systematically evaluated. Untargeted metabolomics was further applied to analyze differential metabolites and enriched metabolic pathways, with the aim of identifying the optimal concentration of quercetin for improving the quality of sex-sorted frozen semen from Australian Wagyu bulls and exploring its potential protective mechanisms from the perspective of metabolic regulation. This study not only contributes to the optimization of cryopreservation systems for bovine sex-sorted frozen semen but also provides theoretical support and metabolomic evidence for the application of natural antioxidants in livestock reproductive biotechnology.

Although the antioxidant effects of quercetin on cryopreserved sperm have been reported in several species, its concentration-dependent effects in sex-sorted Australian Wagyu bull semen and their association with the thawed-semen metabolomic profile remain insufficiently characterized. The present study therefore focuses on defining this concentration response and identifying treatment-associated metabolic pathways rather than presenting quercetin itself as a novel antioxidant.

## 2 Materials and methods

### 2.1 Animals, semen collection, and experimental design

Semen from a total of 5 healthy Australian Wagyu bulls age 34–36 months were collected. All bulls were housed on the Guizhou Bull Station, where the temperature was maintained at approximately 20–25 °C and the humidity was controlled at around 60–70%. They were provided with a standardized diet to ensure consistency in their physiological conditions. Semen was collected from each bull using an artificial vagina at 7-day intervals, with three consecutive collections obtained from each bull. All ejaculates were processed individually. Only ejaculates with sperm motility ≥80% and a sperm abnormality rate ≤ 15% were included in the subsequent experiments. In this study, the five bulls were considered biological replicates, whereas the three independently collected ejaculates from each bull were treated as technical replicates.

Quercetin (purity ≥98%, Sigma-Aldrich, USA) was dissolved in dimethyl sulfoxide (DMSO) and diluted with freezing extender to obtain final concentrations of 0, 10, 50, 100, 150 and 200 μM. The final DMSO concentration was maintained below 0.1% in all groups, including the control. The experimental groups were designated as Q0, Q10, Q50, Q100, Q150, and Q200, respectively.

### 2.2 Sex sorting cryopreservation

Semen cryopreservation and thawing were performed according to previously described procedures (14), with minor modifications. Ejaculates with sperm motility ≥70% were selected for cryopreservation and diluted 1:1 with cryopreservation diluent (15). The diluted semen was incubated at 25 °C for 1 h and subsequently transferred to a programmable cooling system (ZXZ-65L, Guangzhou Zhuxianzi Technology Co., Ltd., China), where it was cooled at 17 °C for 2 h. The samples were then centrifuged at 800 × g for 15 min at 17 °C, and the supernatant was removed. Cryopreservation base solution I (Green Ores, China), supplemented with 20% egg yolk, was added to the sperm pellet, and the samples were equilibrated at 4 °C for 2 h in a refrigerated cabinet (FYL-YS-88L, Beijing Electric Appliance Co., Ltd., China) (15). After equilibration, cryopreservation base solution II containing 6% glycerol (Green Ores, China) was mixed with the cooled semen at a 1:1 ratio. The treated semen was loaded into 0.5-mL straws (Minitüb, Germany), which were positioned 3–4 cm above the surface of liquid nitrogen and exposed to liquid nitrogen vapor for 15 min. The straws were then plunged directly into liquid nitrogen and stored in a liquid nitrogen tank for long-term preservation.

After storage in liquid nitrogen for at least 1 week, the frozen semen straws were thawed in a 50 °C water bath for 16 s. Both ends of each straw were cut, and the thawed semen was transferred into a 5-mL centrifuge tube. The semen was then diluted 1:8 with thawing solution prewarmed to 37 °C and incubated at 37 °C for 15 min. post-thaw sperm quality was subsequently evaluated using the CASA system. Each sample was assessed in triplicate, and the results are presented as the mean ± standard deviation.

### 2.3 Evaluation of sperm quality

Sperm quality parameters were evaluated using CASA as per the World Health Organization standards (14). To evaluate the progressive motility of sperm, 3 μL of fresh semen was immediately evaluated through a computer-assisted sperm analysis system (CASA, Nikon, Eclipse E200, Basler, acA780-75gc, SCA sperm class analyzer) (16). Only semen samples were considered for analysis with a volume ≥ 2.0 mL and revealed ≥ 70% motility. Sperm morphology was evaluated using stained smears under light microscopy, and at least 200 spermatozoa were counted for each sample. Plasma membrane integrity and acrosome integrity were assessed using commercial fluorescence staining kits. Acrosome integrity was determined using FITC-PSA staining. Mitochondrial membrane potential (MMP) was evaluated using a fluorescent probe assay. DNA integrity was determined using the TUNEL assay. For all fluorescence-based assays, at least 200 spermatozoa were analyzed per sample under a fluorescence microscope (Olympus IX73, Japan) (17). Intracellular reactive oxygen species (ROS) levels were measured using DCFH-DA fluorescent staining. The fluorescence intensity was quantified using Image-Pro Plus software. Antioxidant-related parameters, including superoxide dismutase (SOD), catalase (CAT), glutathione peroxidase (GSH-Px), total antioxidant capacity (T-AOC), and malondialdehyde (MDA), were determined using commercial assay kits according to the manufacturers' instructions (18).

### 2.4 Untargeted metabolomics analysis

Based on the sperm quality results, the Q50 and Q0 groups were selected for untargeted metabolomics analysis. Thawed semen samples were centrifuged at 12,000 × g for 10 min at 4 °C, and the supernatants were collected for metabolite extraction.

Because metabolites were measured in post-centrifugation thawed-semen supernatant, the detected profile may include extracellular sperm-derived metabolites, residual seminal components, and freezing-extender constituents. Accordingly, these data are interpreted as treatment-associated alterations in the thawed-semen supernatant metabolome rather than direct evidence of intracellular sperm metabolic remodeling.

Briefly, 200 μL of sample was mixed with 800 μL methanol/acetonitrile (1:1, v/v), vortexed and incubated at 4 °C for protein precipitation. After centrifugation, the supernatant was collected, vacuum-dried, and reconstituted in methanol/water (1:1, v/v). Following filtration through a 0.22-μm membrane, samples were subjected to LC-MS/MS analysis. Quality control (QC) samples were prepared by pooling equal aliquots from all samples.

The Wagyu semen was used for non-targeted metabolomics analysis via UHPLC-QE-Orbitrap/MS (ultra-high-performance liquid chromatography coupled with Q-Exactive Orbitrap mass spectrometry). During metabolite extraction, a 50 mg sperm pellet was first dissolved in 200 μL of water and subjected to three rounds of vortexing using a cell sonicator (Biosafer, USA). A 50 μL aliquot of the homogenate was taken to determine protein concentration using a BCA kit according to the manufacturer's instructions. To the remaining homogenate, 600 μL of solvent (acetonitrile:methanol = 1:1) was added; the mixture was vortexed for 30 s and incubated at −40 °C for 1 h to precipitate proteins. Subsequently, the mixture was centrifuged at 13,800 × g and 4 °C for 15 mis. A 600 μL aliquot of the supernatant was transferred to a new tube for further processing and finally moved to an LC injection vial, where it was stored at −80 °C until analysis. Quality control (QC) samples were prepared by pooling equal volumes of supernatant from all sperm samples. LC-MS/MS analysis was performed using a UHPLC system (Vanquish, Thermo Fisher Scientific, USA) equipped with a UPLC BEH Amide column (2.1 mm × 100 mm, 1.7 μm) coupled to a Q Exactive HFX mass spectrometer (Orbitrap MS, Thermo, USA).

Raw data were processed using MassHunter software for peak extraction, alignment and normalization. Metabolites were identified by comparison with the HMDB, KEGG and Metlin databases. Differential metabolites were screened based on variable importance in projection (VIP) > 1 and P < 0.05.

### 2.5 Statistical analysis

All data are presented as mean ± standard deviation (SD). Statistical analyses were performed using SPSS 26.0 software (IBM Corp., USA). Differences among groups were analyzed using one-way analysis of variance (ANOVA) followed by Duncan's multiple range test. Differences were considered statistically significant at *P* < 0.05.

Metabolomics data were analyzed using SIMCA 16.0 software. Principal component analysis (PCA) and orthogonal partial least squares discriminant analysis (OPLS-DA) were used to evaluate metabolic differences between groups. Kyoto Encyclopedia of Genes and Genomes (KEGG) enrichment analysis was performed to identify significantly altered metabolic pathways.

## 3 Results

### 3.1 Effects of quercetin on the motility of sex-sorted frozen semen from Australian Wagyu bulls

The effects of different concentrations of quercetin on the post-thaw motility of sex-sorted frozen semen from Australian Wagyu bulls are shown in Table 1. Compared with the control group, the 50 μM quercetin-treated group showed superior performance in sperm viability, motility, VSL, VCL, and VAP (*P* < 0.05), with values of 48.68 ± 7.76%, 43.51 ± 6.67%, 16.13 ± 2.91 μm/s, 19.90 ± 4.32 μm/s, and 13.97 ± 3.03 μm/s, respectively. In contrast, sperm viability, motility, and several motility-related parameters were markedly reduced in the 150 and 200 μM groups. Compared with velocity-related parameters, WOB, BCF, LIN, and MAD were less affected by quercetin supplementation. Overall, 50 μM quercetin exerted a relatively pronounced beneficial effect on post-thaw sperm motility, whereas high concentrations of quercetin may adversely affect sperm motility function.

**Table 1:** Effects of quercetin on the motility of sex-sorted frozen semen from Australian Wagyu bulls.

Items	0 μM	10 μM	50 μM	100 μM	150 μM	200 μM
Viability (%)	38.38 ± 5.53^b^	44.24 ± 7.06^ab^	48.68 ± 7.76^a^	35.42 ± 4.69^b^	28.75 ± 4.89^c^	27.98 ± 4.16^c^
Motility (%)	34.35 ± 4.93^c^	38.44 ± 7.06^bc^	43.51 ± 6.67^a^	30.71 ± 3.66^c^	24.48 ± 4.83^d^	23.67 ± 2.96^d^
VSL (μm/s)	13.42 ± 2.86^b^	15.71 ± 5.73^ab^	16.13 ± 2.91^a^	12.12 ± 3.05^b^	10.11 ± 2.75^b^	9.58 ± 1.84^b^
VCL (μm/s)	16.10 ± 3.19^b^	18.78 ± 6.85^ab^	19.90 ± 4.32^a^	14.49 ± 3.55^b^	12.21 ± 3.12^b^	11.42 ± 1.99^b^
VAP (μm/s)	11.30 ± 2.24^b^	13.18 ± 4.81^ab^	13.97 ± 3.03^a^	10.18 ± 2.49^b^	8.57 ± 2.19^b^	8.02 ± 1.40^b^
ALH (μm)	4.80 ± 0.95^b^	5.60 ± 2.04^ab^	5.93 ± 1.29^a^	4.32 ± 1.06^b^	3.64 ± 0.93^b^	3.40 ± 0.59^b^
WOB	0.20 ± 0.08	0.22 ± 0.06	0.26 ± 0.09	0.19 ± 0.08	0.18 ± 0.09	0.17 ± 0.08
BCF	4.65 ± 1.94	5.47 ± 1.25	5.42 ± 1.33	4.65 ± 1.71	3.44 ± 1.37	4.17 ± 0.64
LIN	0.83 ± 0.04	0.83 ± 0.04	0.81 ± 0.05	0.83 ± 0.03	0.81 ± 0.07	0.83 ± 0.04
MAD	5.74 ± 2.66	6.70 ± 1.80	6.76 ± 1.72	5.65 ± 2.23	4.26 ± 1.68	5.01 ± 0.71

VSL, straight-line velocity; VCL, curvilinear velocity; VAP, average path velocity; ALH, amplitude of lateral head displacement; WOB, wobble; BCF, beat cross frequency; LIN, linearity; MAD, mean angular displacement.

Different lowercase letters within the same row indicate significant differences (*P* < 0.05). Rows without letter annotations indicate no significant differences among groups. Data are presented as mean ± SD.

### 3.2 Effects of quercetin on sperm abnormality rate, plasma membrane integrity, acrosome integrity, and mitochondrial membrane potential

As shown in Figure 1, there was no significant difference in sperm abnormality rate among the groups treated with different concentrations of quercetin, indicating that quercetin treatment did not markedly alter the proportion of morphologically abnormal sperm after thawing. The plasma membrane integrity and acrosome integrity in the 50 μM quercetin-treated group were higher than those in the other groups (*P* < 0.05), suggesting that an appropriate concentration of quercetin helps maintain sperm membrane structural stability. In contrast, the 150 and 200 μM groups showed lower plasma membrane integrity and acrosome integrity, indicating that high concentrations of quercetin may weaken its protective effect.

**Figure 1:**
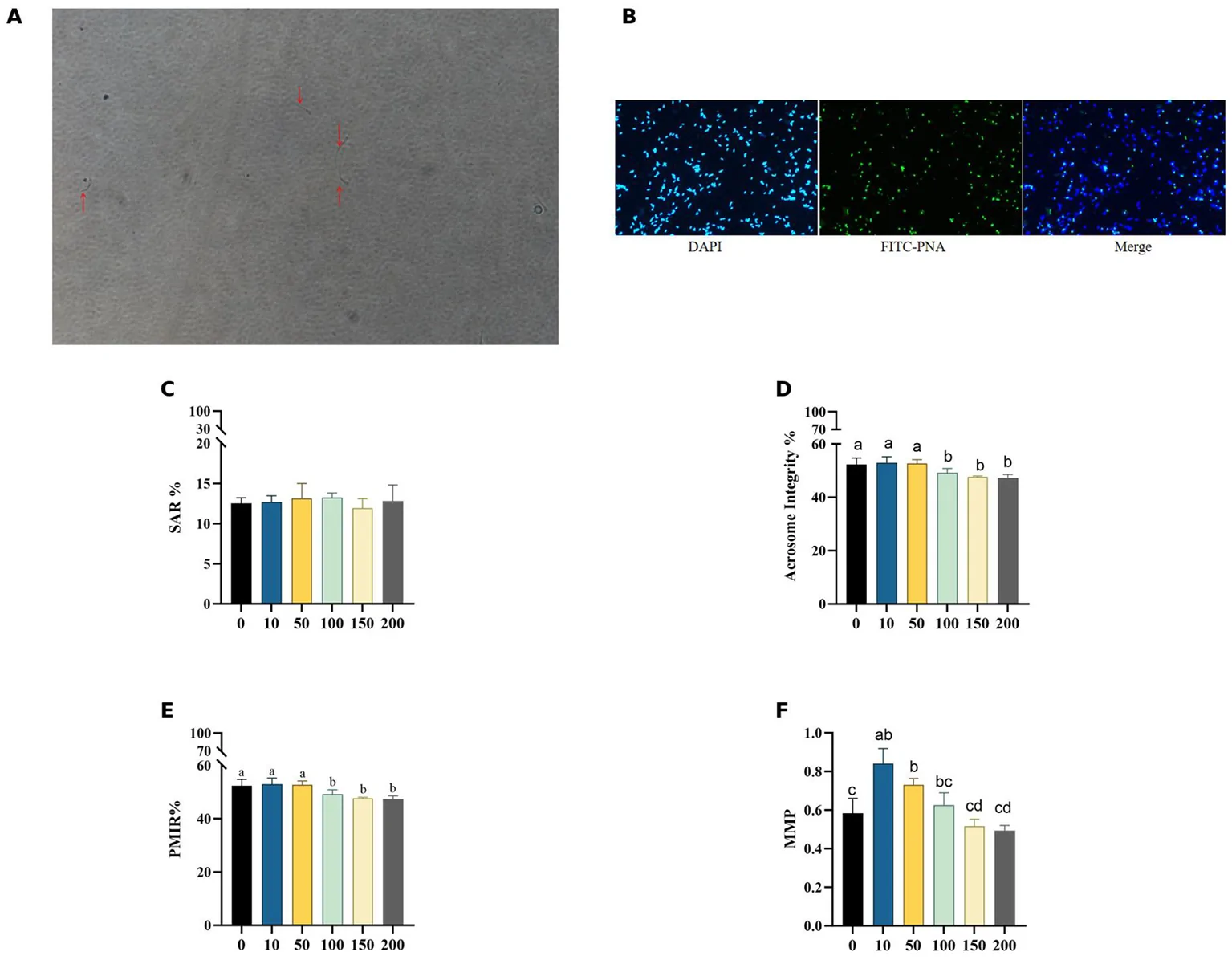
Effects of different quercetin concentrations on sperm morphology and functional parameters in sex-sorted frozen semen from Australian Wagyu bulls. **(A)** Plasma membrane integrity micrograph; **(B)** representative acrosome integrity fluorescence images; **(C)** sperm abnormality rate; **(D)** acrosome integrity rate; **(E)** plasma membrane integrity rate; and **(F)** mitochondrial membrane potential. Different lowercase letters indicate significant differences among groups (*P* < 0.05). Data are presented as mean ± SD.

Mitochondrial membrane potential differed significantly among the different concentration groups (*P* < 0.05). The 10 μM group showed the highest MMP, whereas MMP gradually decreased with increasing quercetin concentration, reaching relatively low levels in the 150 and 200 μM groups. These results indicate that different sperm functional parameters do not respond uniformly to quercetin concentration. The 50 μM group showed better overall sperm quality, whereas the 10 μM group was more favorable for maintaining mitochondrial membrane potential. The results are shown in Figures 1A–F.

### 3.3 Effects of quercetin on sperm ROS levels and antioxidant-related indices

In the cryopreservation system for sex-sorted frozen semen from Australian Wagyu bulls, quercetin supplementation reduced post-thaw sperm ROS levels (P < 0.05). Among the treatment groups, the 50 μM quercetin group showed the lowest ROS level, indicating that this concentration was effective in alleviating oxidative stress induced by freezing and thawing. Consistent with the changes in ROS levels, the activities of antioxidant enzymes, including SOD, CAT, and GSH-Px, were increased in the 50 μM quercetin group, whereas MDA levels were reduced and T-AOC levels were increased (*P* < 0.05; Figure 2). These results suggest that an appropriate concentration of quercetin may improve the post-thaw quality of sex-sorted frozen semen by enhancing antioxidant defense capacity and reducing lipid peroxidation.

**Figure 2:**
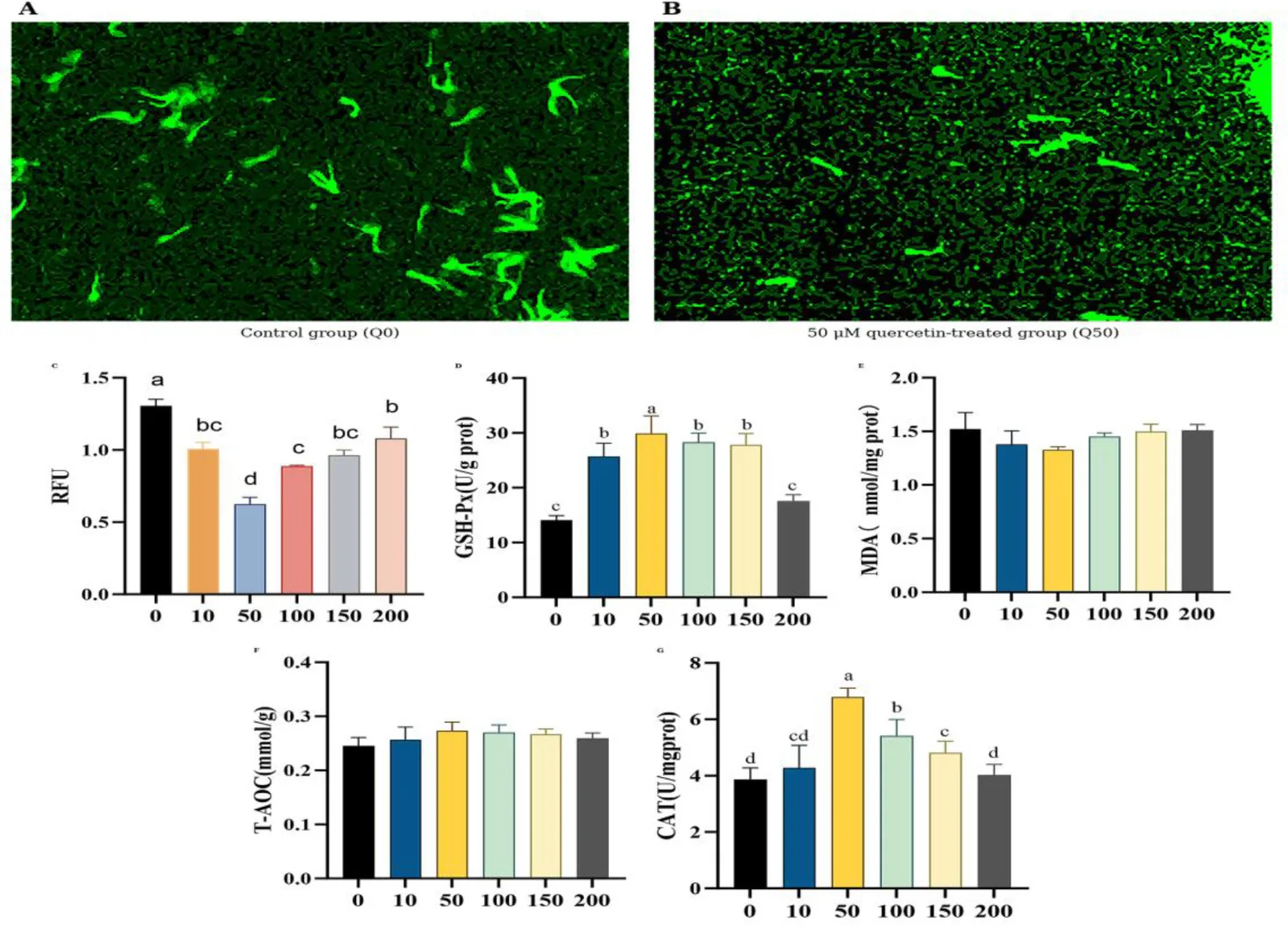
Effects of quercetin on reactive oxygen species (ROS) levels and antioxidant-related indices in sex-sorted frozen semen from Australian Wagyu bulls. Representative DCFH-DA fluorescence images are shown for the control (Q0) and 50 μM quercetin (Q50) groups, together with quantitative ROS, glutathione peroxidase (GSH-Px), malondialdehyde (MDA), total antioxidant capacity (T-AOC), and catalase (CAT) results across the six quercetin concentrations. Different lowercase letters indicate significant differences among groups (*P* < 0.05). Data are presented as mean ± SD.

### 3.4 Effects of quercetin on sperm DNA integrity

As shown in Figure 3, different concentrations of quercetin had significant effects on sperm DNA integrity in sex-sorted frozen semen from Australian Wagyu bulls (*P* < 0.05). The 50 μM group showed a relatively high DNA integrity rate, which was significantly higher than those in the 100, 150, and 200 μM groups (*P* < 0.05), but did not differ significantly from the 0 and 10 μM groups. As quercetin concentration continued to increase, DNA integrity gradually decreased, with the 150 and 200 μM groups showing significantly lower DNA integrity than the control group (*P* < 0.05). Therefore, an appropriate concentration of quercetin helps maintain DNA integrity, whereas excessive concentrations may increase the risk of DNA damage.

**Figure 3:**
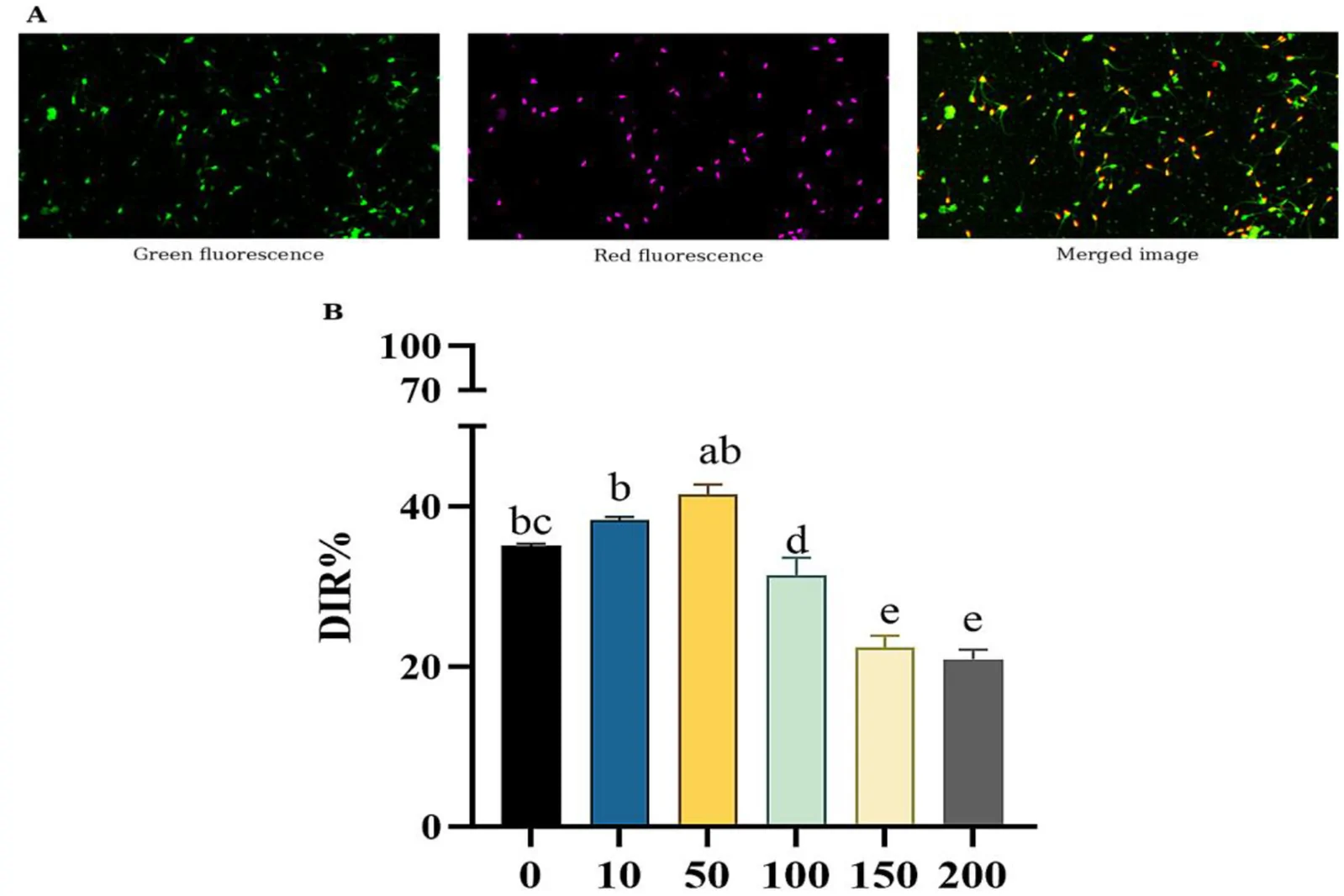
Effects of quercetin on sperm DNA integrity in sex-sorted frozen semen from Australian Wagyu bulls. Representative TUNEL fluorescence images and the DNA integrity rate (DIR) are shown. Different lowercase letters indicate significant differences among groups (*P* < 0.05). Data are presented as mean ± SD.

### 3.5 Metabolomics analysis

#### 3.5.1 PCA and OPLS-DA analyses

To characterize metabolic differences associated with the treatment showing the most favorable overall sperm-quality profile, metabolomics analysis was performed on the Q50 and Q0 groups. PCA showed a certain separation trend in the overall metabolite distribution between the Q50 and Q0 groups, indicating that treatment with 50 μM quercetin may alter the metabolic profile of thawed-semen samples. OPLS-DA further demonstrated differences in metabolic characteristics between the two groups (Figure 4).

**Figure 4:**
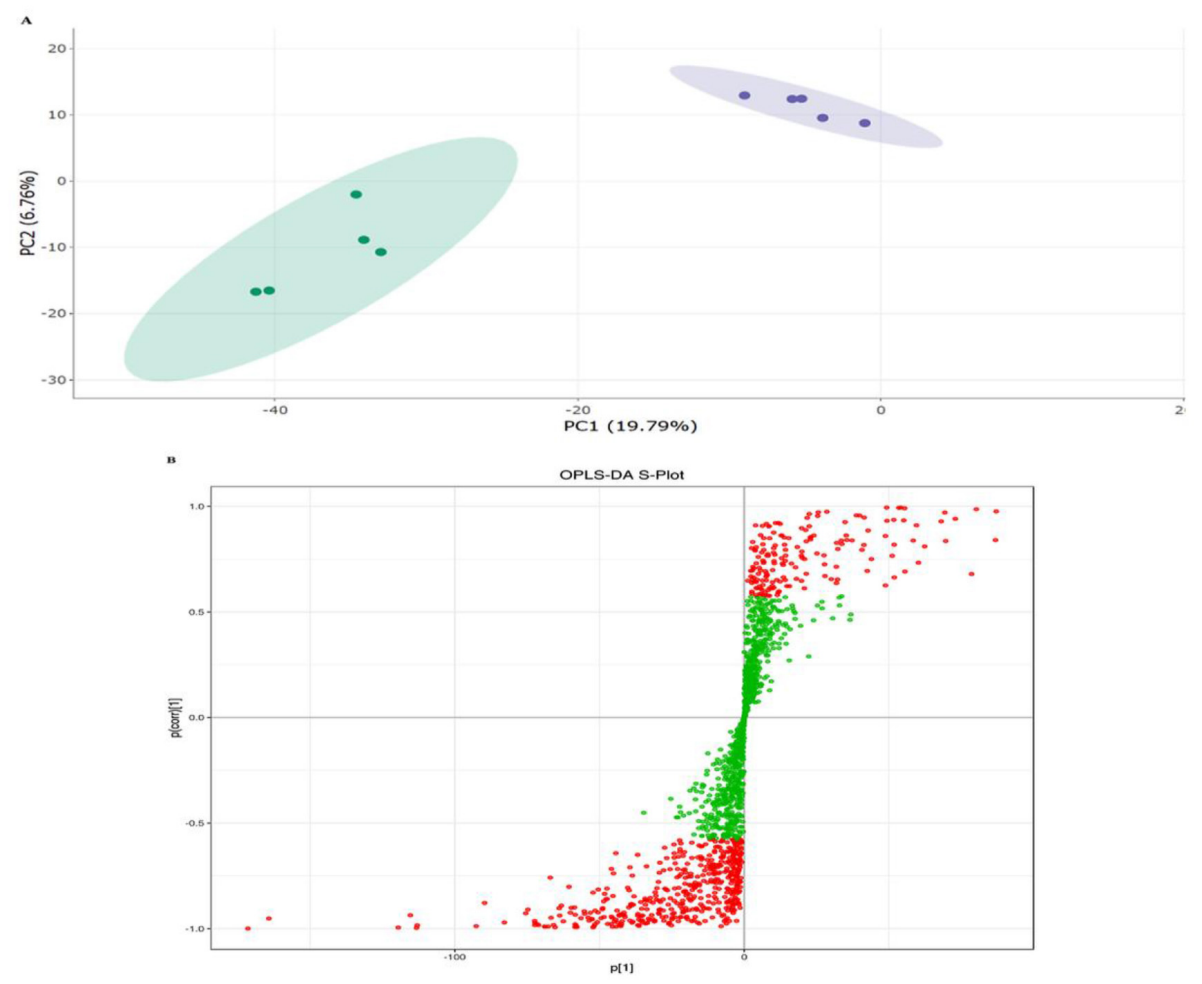
Multivariate analysis of metabolomics data from the Q50 and Q0 groups. The principal component analysis score plot is shown above, and the orthogonal partial least squares discriminant analysis S-plot is shown below.

#### 3.5.2 Screening of differential metabolites

Based on the screening criteria of VIP > 1 and *P* < 0.05, a total of 526 differential metabolites were identified between the Q50 and Q0 groups, including 131 significantly upregulated and 395 significantly downregulated metabolites (Figure 5 and Supplementary Table S1). The volcano plot showed that multiple metabolites in frozen semen samples changed significantly after quercetin treatment, indicating that 50 μM quercetin may regulate the metabolic status of frozen semen samples.

**Figure 5:**
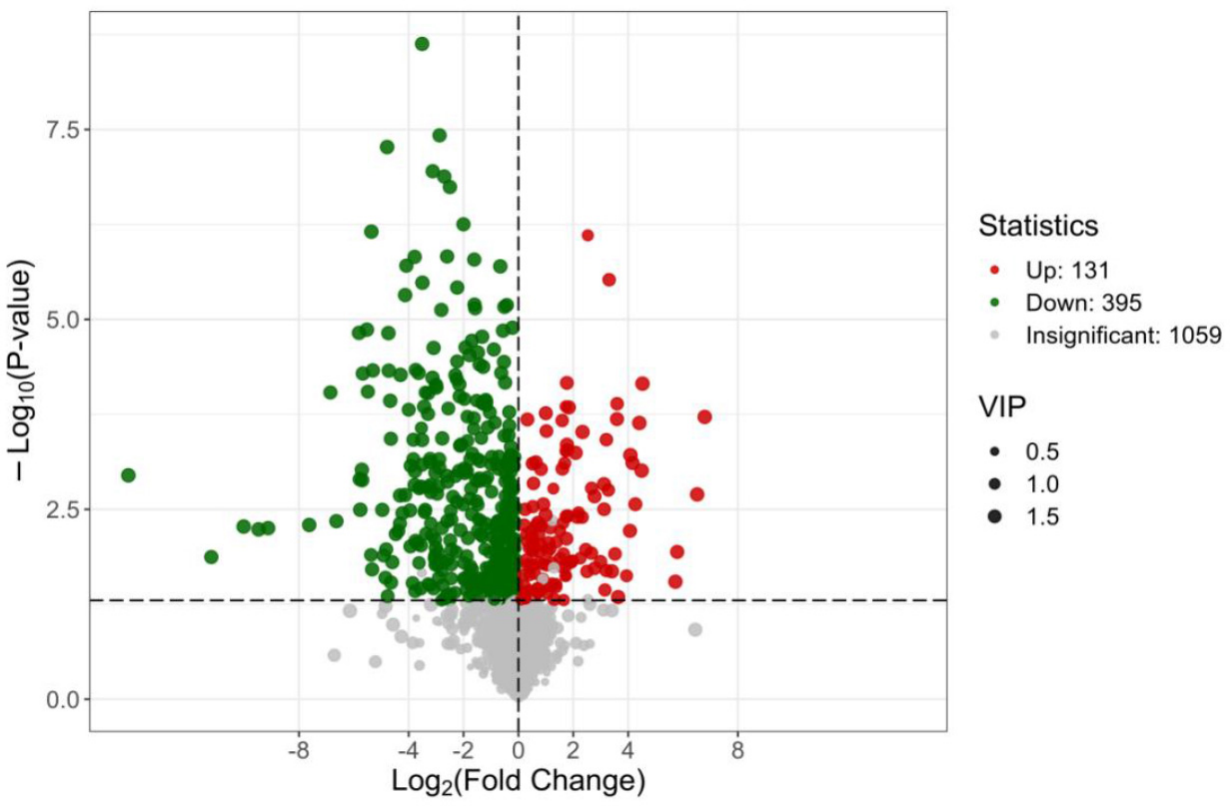
Volcano plot of differential metabolites between the Q50 and Q0 groups. Differential metabolites were screened using VIP > 1 and *P* < 0.05; red and green points denote upregulated and downregulated metabolites, respectively, and gray points denote non-significant metabolites.

#### 3.5.3 KEGG pathway enrichment analysis

KEGG enrichment analysis showed that the differential metabolites between the Q50 and Q0 groups were mainly enriched in lipid-related pathways, including glycerophospholipid metabolism, arachidonic acid metabolism, choline metabolism, biosynthesis of unsaturated fatty acids, and cholesterol metabolism. Among these, glycerophospholipid metabolism showed a relatively high enrichment degree and involved a larger number of differential metabolites, indicating that lipid metabolic remodeling may be one of the major metabolic features through which quercetin affects the quality of sex-sorted frozen semen (Figure 6 and Supplementary Table S1). These results are consistent with the observed reduction in ROS levels, increased antioxidant enzyme activities, and improved plasma membrane and acrosome integrity, suggesting that 50 μM quercetin may alleviate sperm cryoinjury by regulating lipid metabolism, reducing the risk of membrane lipid peroxidation, and maintaining membrane structural stability. Because metabolomics was limited to the Q50-vs.-Q0 comparison, these pathway changes should be interpreted as treatment-associated differences and not as a demonstrated concentration-dependent or intracellular causal mechanism.

**Figure 6:**
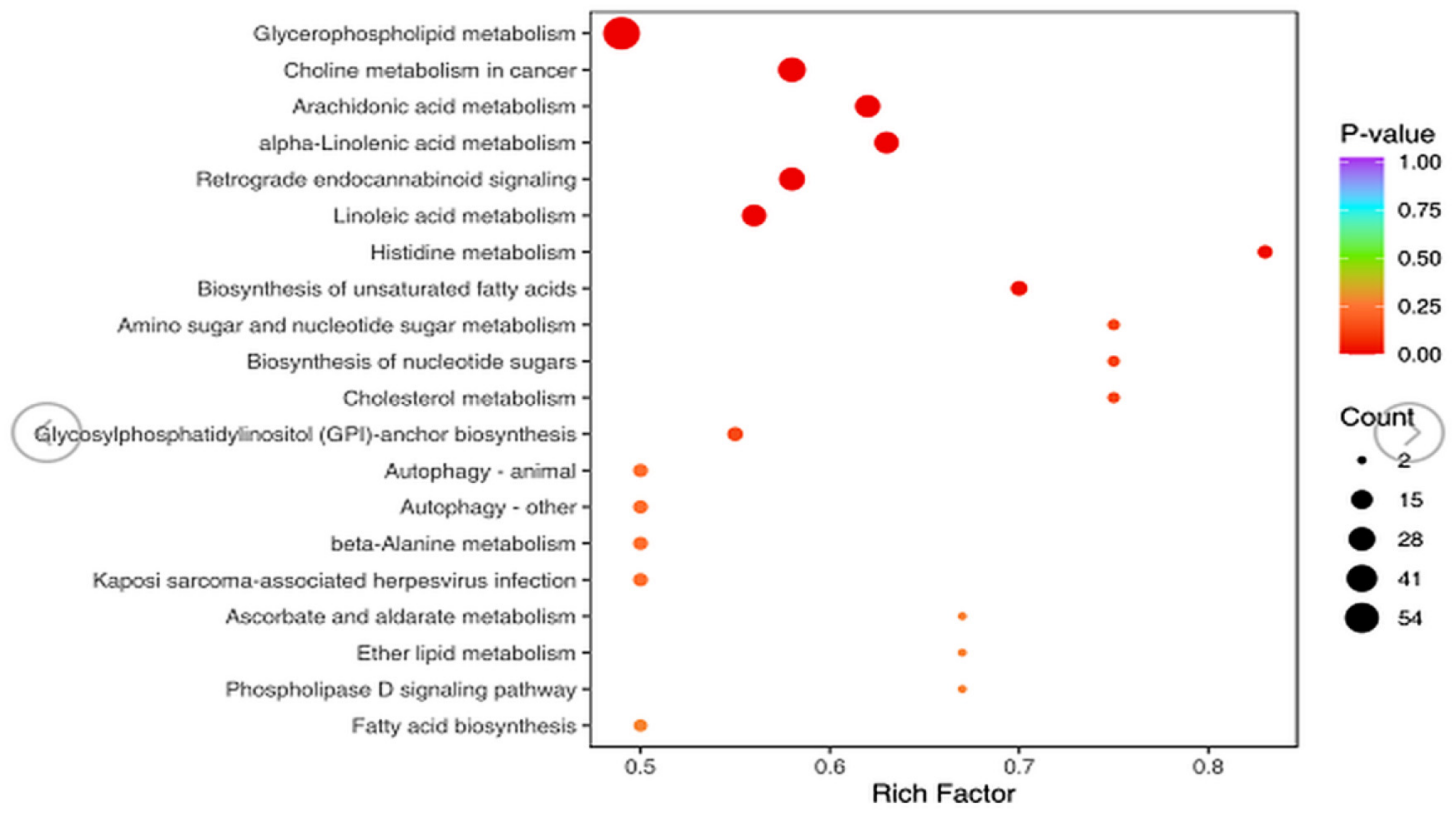
Kyoto Encyclopedia of genes and genomes pathway enrichment analysis of differential metabolites between the Q50 and Q0 groups. Bubble size represents the number of differential metabolites, and bubble color represents the enrichment *P*-value.

#### 3.5.4 Clustering analysis of differential metabolites

The heatmap of differential metabolites showed clear differences in metabolite expression patterns between the Q50 and Q0 groups (Figure 7). Several lipid-related metabolites were downregulated in the Q50 group, suggesting that quercetin treatment may reduce the accumulation of certain lipid peroxidation substrates or lipid stress-related metabolites. A small number of upregulated metabolites may be associated with antioxidant defense or membrane lipid remodeling.

**Figure 7:**
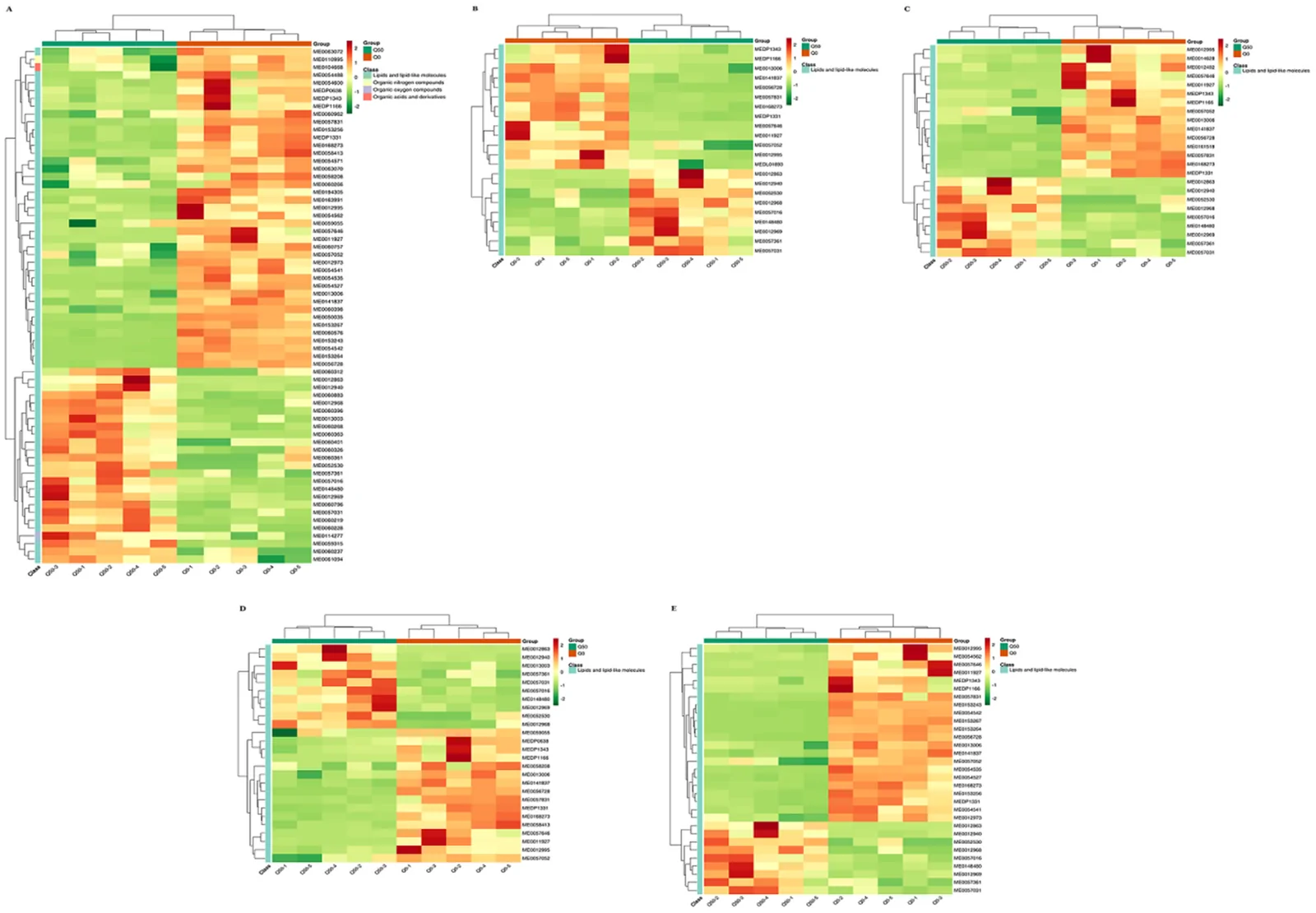
Hierarchical clustering heatmaps of differential metabolites between the Q50 and Q0 groups. Metabolite identifiers and group annotations should be verified against the final metabolite identification table before submission.

## 4 Discussion

The present study demonstrated that quercetin exerted concentration-dependent effects on the post-thaw quality of sex-sorted frozen semen from Australian Wagyu bulls. Among the tested concentrations, 50 μM quercetin showed the most favorable overall effect, as reflected by improved sperm viability, motility, VSL, VCL, and VAP. These findings indicate that an appropriate concentration of quercetin can enhance the motility performance of sex-sorted frozen-thawed sperm. Previous studies have also shown that quercetin improves post-thaw sperm quality in different species through free radical scavenging and mitochondrial protection (19–22). However, when the concentration of quercetin increased to 150 and 200 μM, sperm viability, motility, and several motility parameters decreased, suggesting that the protective effect of quercetin has a specific concentration window. Excessive quercetin supplementation may weaken its antioxidant benefits or even increase the risk of oxidative injury (23, 24). This inference requires further verification through the detection of GSH depletion, lipid peroxidation chain reactions, or quercetin-derived oxidative intermediates.

Cryopreservation-induced oxidative stress is considered one of the principal causes of sperm dysfunction following freezing and thawing. During cryopreservation, excessive accumulation of ROS disrupts membrane lipids, impairs mitochondrial activity, and induces oxidative DNA damage, ultimately reducing sperm fertilizing capacity (6, 25–27). In the present study, supplementation with 50 μM quercetin significantly improved sperm motility parameters, including VSL, VCL, and VAP, while simultaneously reducing intracellular ROS levels. These results are consistent with previous reports in boar, stallion, goat, and buffalo sperm, where quercetin supplementation enhanced post-thaw sperm kinetics and antioxidant status (8, 9, 20, 28). The improvement in sperm motility observed here is likely associated with the free radical scavenging activity of quercetin and its ability to preserve mitochondrial function under cryogenic stress conditions.

Notably, the protective effect of quercetin exhibited a clear concentration-dependent pattern. While moderate concentrations improved sperm function, higher concentrations (150–200 μM) negatively affected motility and several functional parameters. Similar biphasic effects have been reported in mammalian reproductive cells, where excessive flavonoid supplementation induced pro-oxidant activity or interfered with cellular redox homeostasis (23). Quercetin can undergo auto-oxidation under certain conditions, generating semiquinone intermediates that may exacerbate oxidative injury when antioxidant buffering capacity is exceeded. Therefore, the beneficial effect of quercetin appears to rely on maintaining an appropriate balance between ROS scavenging and physiological redox signaling. This concentration-dependent response may partially explain inconsistencies among previous studies regarding the optimal supplementation level of quercetin in semen extenders.

Mitochondrial function plays a pivotal role in ATP production and sperm motility. Excessive ROS accumulation disrupts mitochondrial membrane integrity and electron transport chain activity, resulting in decreased MMP and impaired energy metabolism (27, 29–31). Interestingly, the present study found that the highest MMP value occurred in the 10 μM group, whereas the best overall sperm quality was observed in the 50 μM group. This discrepancy indicates that individual sperm functional parameters may respond differently to antioxidant supplementation. Lower concentrations of quercetin may preferentially preserve mitochondrial membrane stability, whereas moderate concentrations provide broader protection against oxidative and membrane damage. Similar differential responses between mitochondrial activity and overall sperm quality have been observed in bovine and porcine cryopreservation studies (27, 29). These findings highlight the complexity of redox regulation in sperm physiology and suggest that optimal antioxidant supplementation should be evaluated comprehensively rather than relying on a single functional endpoint.

Sperm plasma membrane and acrosome integrity are essential for maintaining sperm viability and fertilization competence. The sperm membrane is highly susceptible to lipid peroxidation because of its elevated content of polyunsaturated fatty acids. In the present study, the Q50 group exhibited higher plasma membrane integrity and acrosome integrity than the high-concentration groups, coinciding with lower ROS levels and elevated antioxidant enzyme activities. These observations support the hypothesis that quercetin stabilizes sperm membrane structure by attenuating oxidative damage during cryopreservation. Previous studies in buffalo and equine semen similarly demonstrated that quercetin supplementation preserved membrane functionality and reduced cryodamage-associated enzyme leakage (19, 28). The current findings further extend these observations to sex-sorted bovine semen, which is particularly vulnerable to membrane destabilization due to the additional mechanical and laser stress imposed during flow cytometric sorting.

DNA integrity is another critical determinant of sperm fertilizing ability and embryo developmental competence (32). Oxidative stress-induced DNA fragmentation has been strongly associated with reduced conception rates and impaired embryo quality. In the present study, moderate quercetin supplementation maintained higher DNA integrity, whereas high concentrations significantly increased DNA damage. These results are consistent with previous findings in human and stallion sperm, where quercetin reduced cryopreservation-associated DNA fragmentation by suppressing oxidative injury (21, 22). The maintenance of DNA integrity observed here is likely linked to reduced ROS accumulation and improved antioxidant defense capacity. Importantly, the Q50 group did not differ significantly from the control and Q10 groups in some DNA-related indices, suggesting that the primary benefit of quercetin lies in preventing excessive cryodamage rather than dramatically enhancing baseline sperm genomic stability. This distinction is important for accurately interpreting the biological significance of antioxidant supplementation.

Untargeted metabolomics further revealed substantial metabolic alterations associated with quercetin supplementation. Differential metabolites were primarily enriched in glycerophospholipid metabolism, arachidonic acid metabolism, unsaturated fatty acid biosynthesis, and cholesterol-related pathways. These pathways are closely associated with membrane fluidity, oxidative susceptibility, and sperm cryoresistance. Glycerophospholipids constitute the structural framework of sperm membranes, and alterations in phospholipid composition directly influence membrane permeability and cryostability. Arachidonic acid metabolism is also strongly linked to oxidative stress and inflammatory lipid signaling. The observed enrichment of these pathways suggests that quercetin may alleviate cryoinjury by modulating membrane lipid remodeling and reducing lipid peroxidation. Previous metabolomic studies have identified lipid metabolism as a major determinant of sperm freezability and fertility in bulls (11, 13). However, few studies have connected antioxidant supplementation with metabolomic remodeling in sex-sorted bovine sperm. Therefore, the present study provides new mechanistic evidence linking antioxidant-mediated cryoprotection with metabolic regulation. However, because the analyzed matrix was thawed-semen supernatant, the metabolite source cannot be assigned exclusively to sperm cells.

Another important implication of this study is the potential application of quercetin in improving the efficiency of commercial sex-sorted semen production. Sex-sorted semen typically exhibits lower fertility than conventional frozen semen because the sorting process itself imposes oxidative and mechanical stress on spermatozoa. By improving post-thaw sperm quality and maintaining membrane and DNA integrity, quercetin supplementation may contribute to enhancing the practical fertility performance of sex-sorted semen in cattle breeding systems. Given the increasing global demand for precision breeding and genetic resource optimization, improving the cryosurvival of sex-sorted semen has considerable economic and reproductive significance for the livestock industry.

Several limitations of this study should also be acknowledged. First, the sample size was relatively limited and derived from a single cattle population, which may restrict the generalizability of the findings across breeds or management systems. Second, the metabolomic analysis primarily provided associative evidence, and the specific molecular targets of quercetin within lipid metabolic pathways remain unclear. Third, fertility outcomes such as *in vivo* conception rate and embryo development were not evaluated. Therefore, the biological relevance of the observed improvements in sperm quality should be further validated under field breeding conditions. In addition, several differential metabolites identified in the metabolomic analysis require further structural confirmation and targeted validation. Future studies integrating targeted lipidomics, mitochondrial functional assays, transcriptomics, and artificial insemination trials would provide a more comprehensive understanding of the molecular mechanisms underlying quercetin-mediated cryoprotection. In addition, metabolomic profiling was restricted to Q50 and Q0; therefore, the present data do not establish a dose-dependent metabolic response or demonstrate that the identified metabolites mediate the functional improvements.

## 5 Conclusion

In conclusion, among the concentrations tested, 50 μM quercetin produced the most favorable overall post-thaw sperm-quality profile, with improved viability and motility-related parameters, reduced ROS, and enhanced antioxidant-related indices. Metabolomic comparison of Q50 and Q0 further identified treatment-associated alterations enriched in lipid-related pathways. These findings support an association between appropriate quercetin supplementation, reduced oxidative stress, and improved post-thaw semen quality, but do not establish enhanced fertilizing capacity.

## Data Availability

The original contributions presented in the study are included in the article/Supplementary material, further inquiries can be directed to the corresponding authors.
